# Correlation between mandibular third molar and mandibular incisor crowding: A retrospective CBCT-based study

**DOI:** 10.34172/joddd.2021.040

**Published:** 2021-12-05

**Authors:** Seerab Husain, Sri Rengalakshmi

**Affiliations:** Department of Orthodontics and Dentofacial Orthopedics, Saveetha Dental College and Hospital, Chennai, India

**Keywords:** Cone-beam computed, tomography, Crowding, Incisor, Malocclusion, Third molar

## Abstract

**Background.** Late mandibular incisor crowding is a fairly common phenomenon experienced by growing adults. The etiology of late mandibular incisor crowding, however, is controversial and inconclusive. Hence, this study aimed to investigate the correlation between mandibular third molar and mandibular incisor crowding using Cone-beam computed tomography (CBCT) data.

**Methods.** The study consisted of 40 samples of CBCT images divided into two groups (n=20). Group A comprised CBCT images without third molars, and group B included CBCT images with third molars. The images were observed in the axial view and manually marked to calculate the amount of crowding using Little’s irregularity index. The obtained values were statistically analyzed using Pearson’s correlation test. SPSS 23 was used for statistical analysis.

**Results.** The results showed a positive correlation between the mandibular third molars and mandibular incisor crowding, which was significant (*P* = 0.033). The mean Little’s irregularity index score for groups A and B were 4.26 and 6.799, respectively (*P* = 0.033).

**Conclusion.** The positive correlation between the two groups suggests an association between the mandibular third molars and mandibular incisor crowding.

## Introduction

Malocclusion is one of the most commonly encountered abnormalities in human dentition. It is the malalignment of the teeth relative to each other and with the surrounding structures. Malocclusion can arise due to various etiological factors and is not related to the teeth only. Any deviation in the skeletal and soft tissue integrity can lead to malocclusion. Malocclusion is more of a developmental disturbance rather than a disease.^1^ It can manifest in all three planes of spaces: transverse, sagittal, and vertical dimensions.

Understanding the etiology of many malocclusion forms is still a dilemma, and it is not easy to pinpoint one specific etiology for several malocclusions accurately.^2^ One such malocclusion is the crowding of teeth. Malocclusion as such is generally synonymous with crowding. Crowding is the deviation in the alignment of the teeth within the same arch. The cause of late incisor crowding is controversial, and the debate about the involvement of third molars in the development of late incisor crowding is still a topic of interest.

Diagnostic aids and imaging modalities in dentistry have undergone a tremendous revolution over the past couple of years. Ever since Wilhelm Röntgen discovered x-rays in 1895, there has been no turning back in terms of development in diagnostic medicine.^3^ Broadbent’s introduction of lateral cephalograms in dentistry opened up new horizons for developments in orthodontic diagnosis, which was the much-required pedestal for innovations in future imaging and diagnostic aids.^4^ Currently, CBCT (cone-beam computed tomography) is widely used in the field of orthodontics for diagnosis and treatment planning of complex cases, such as cases involving asymmetries, pathologies, tooth impactions, cleft of lip/palate, etc.^5^

The CBCT technique relies on the principles of tomosynthesis.^6^ The CBCT scan involves an x-ray source and an opposing x-ray sensor that rotates around the patient’s head to record a series of images in the form of slices called voxels. These slices are then reconstructed into a three-dimensional image using mathematical algorithms.^7^ CBCT produces less radiation exposure than the CT scan technique and is hence ideal as an imaging modality in dentistry. Since impacted teeth can easily be visualized on a CBCT image along with the normal dentition, the records obtained from this imaging modality were selected for this study.

This study aimed to evaluate the correlation between the presence and absence of mandibular third molars and the mandibular lower incisor crowding using CBCT images.

## Methods

This CBCT-based study consisted of a retrospective collection of 40 CBCT images. The CBCT images were retrospectively obtained from the Saveetha Institute of Medical and Technical Sciences (SIMATS). These images were divided into two groups: A, in which the 3rd molars were absent, and B, in which the 3rd molars were present, with 20 images in each group. Ethical approval was obtained from the institutional review board.

### The inclusion criteria

- Class I malocclusion
- CBCT images of patients in the 18-30 age group
- CBCT images recorded in the database of the University
- CBCT images with the presence of all the permanent dentition

### The exclusion criteria

- Presence of impacted teeth
- Presence of deciduous teeth
- Presence of skeletal asymmetries
- Presence of any underlying pathologies
- Partial or complete absence of teeth, other than third molars

The CBCT images were reviewed using the Sirona Galileos Viewer software (Bensheim, Germany). The samples were categorized into the two study groups by looking for the presence/absence of mandibular third molar on the panoramic view. After categorization, the amount of crowding was calculated using Little’s irregularity index. The index consisted of the following scores^8^:

- 0: Perfect alignment
- 1-3: Minimal irregularity
- 4-6: Moderate irregularity
- 7-9: Severe irregularity
- >10: Very severe irregularity

**Figure 1:**
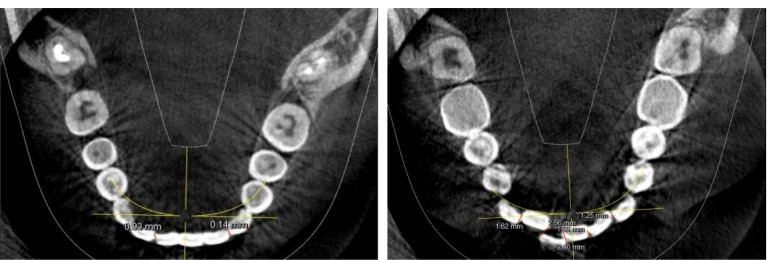
Measurement of Little’s irregularity index using CBCT axial view.

The images were viewed from the axial dimension to calculate mandibular incisor crowding. The images were adjusted in the axial view to the point where the incisal edges and the contact points of mandibular incisors were barely visible. The points were manually plotted from the mesial incisal edge of one tooth to the distal incisal edge of another tooth to make linear measurements. Such points were plotted from the mesial incisal edge of the left mandibular canine to the mesial incisal edge of the right mandibular canine, as shown in (Figure 1). The linear measurements were summed up to obtain Little’s irregularity index score for that sample. The obtained values were tabulated and subjected to statistical analysis. SPSS 23 was used for statistical analysis. Descriptive statistics and Pearson’s correlation test were performed to determine the correlation between mandibular third molars and mandibular lower incisor crowding.

## Results

These descriptive statistics showed that the mean and standard deviation in group A was 4.26 ± 4.88 mm, categorized as moderate irregularity. Group B had a mean score of 6.79 ± 5.45 mm, which indicated severe irregularity (Table 1). The results for Pearson’s correlation test are presented in Table 2. There was a positive correlation between groups A and B, which was statistically significant *P* = 0.033), indicating an association between the presence of mandibular third molar and mandibular incisor crowding.

**Table 1:** Descriptive statistics showing the means and standard deviations of Little’s irregularity index scores

**Sample**	**Group A** **(without third molars)**	**Group B** **(with third molars)**
1	0.83	4.93
2	3.47	19.80
3	0.00	9.24
4	7.63	4.00
5	0.60	6.33
6	20.25	20.02
7	0.30	7.48
8	6.90	4.65
9	0.00	5.09
10	6.41	3.05
11	2.50	5.92
12	2.00	2.30
13	1.11	1.38
14	3.37	4.86
15	1.32	1.51
16	3.72	5.21
17	1.68	1.10
18	4.10	14.62
19	7.60	8.10
20	11.41	6.39
Mean ± SD	4.26 ± 4.88	6.799 ± 5.45

**Table 2:** Pearson’s correlation test showing a positive correlation between the two groups, which was statistically significant (*P* < 0.05)

		**Without the third molar**	**With the third molar**
Without the third molar	Pearson’s correlation	1	0.479^*^
	Sig. (2-tailed)		0.033
	N	20	20
With the third molar	Pearson’s correlation	0.479^*^	1
	Sig. (2-tailed)	0.033	
	N	20	20

*Correlation is significant at the 0.05 level (2-tailed).

## Discussion

The impact and association of third molars in the occurrence of late incisor crowding have been a topic of interest for orthodontists and oral surgeons alike. According to a study by Lindauer et al,^9^ orthodontists believe that third molars are not responsible for incisor crowding. In contrast, surgeons believe that the third molars are responsible for late incisor crowding, advocating the prophylactic extraction of the third molars for this very reason.^9^ A similar survey between American and Swedish orthodontists showed that both believed that the erupting third molars exerted an anterior force. They were also believed that they rarely or never caused crowding.^10^

Ades et al^11^ reported an increase in the incisor irregularity and decreased arch length and intercanine width with aging. However, the role of the third molar in this event is not significant, and it does not necessitate the extraction of third molars for the sake of retention.^11^ Niedzielska^12^ used Ganss ratio and measured the crowding and arch length to determine an association between third molar and incisor crowding and supported the third molar extraction.^12^ Lindqvist and Thilander^13^ reported 70% crowding on the side of the arch with a third molar compared to the side without a third molar.

Some studies have disproved this theory on the association between third molars and late incisor crowding. Bishara et al^14^ and Singh & Shivaprakash^15^ discussed how various literature showed contradictory results on the association between third molars and incisor crowding, concluding that there is no conclusive evidence to show an association between third molars and incisor crowding. A randomized controlled clinical trial by Harradine et al^16^ showed that the difference in the Little’s irregularity index, intercanine width, and arch length of the subjects with and without third molars were not statistically significant and did not justify the extraction of third molars.^16^ Shah et al^17^ used modified arch analysis by Lundstrom to calculate anterior crowding and concluded that the results were insignificant and could not justify the extraction of third molars to prevent incisor crowding.

This study showed a positive correlation between mandibular third molar and mandibular incisor crowding, which might be attributed to the force exerted by the forward drift of second molars and the mesial force exerted by erupting third molars.^18^ Although this study showed a positive correlation between the two, it is important to note other factors like age, arch length, jaw size, tooth dimension, soft tissue pressure, etc., on incisor crowding. There is a decrease in the arch size, inter-canine width, and an increase in the tooth dimension in males and females with aging, which could also contribute to late incisor crowding.^19^ Similarly, jaw size also plays an important role in the development of crowding. Patients with Class II skeletal patterns with a smaller mandible tend to have more crowding than the normal counterparts.^20,21^ Periodontal tissues have also been reported to influence late incisor crowding by its forces exerted to maintain tight interproximal contacts.^22^

Crowding is a complex phenomenon with a multifactorial etiology. This, however, does not rule out the effect of third molars on lower incisor crowding. This study used CBCT imaging to show an association between mandibular third molars and mandibular incisor crowding.

## Conclusion

Within the limitations of this study, it can be concluded that there is a positive correlation between the mandibular third molar and mandibular incisor crowding, establishing the role of third molars as one of the etiological factors for crowding if not the only one. Further prospective longitudinal studies on their association with larger sample sizes would provide more concrete evidence to establish a correlation between crowding and third molars. It is also important to understand the other etiological factors behind crowding as it could help us formulate a viable treatment plan and intercept crowding at the right time to reduce unwanted loss of time to treat an otherwise avoidable circumstance.

## Authors’ Contributions

Both authors equally contributed to the acquisition, analysis, and interpretation of data for the work and drafting and revising the work.

## Acknowledgments

This study was supported by the database provided by the Saveetha Institute of Medical and Technical Sciences (SIMATS). We thank Dr. Aravind Kumar, the anonymous referees/reviewers for their useful suggestions, and our colleagues for their support and encouragement throughout this study.

## Funding

The study was funded by Saveetha Institute of Medical and Technical Sciences (SIMATS).

## Competing Interests

The authors declare that there is no conflict of interest.

## Ethics Approval

Ethical approval was obtained from the institutional review board of Saveetha Institute of Medical and Technical Sciences (SIMATS).
